# Did the COVID-19 pandemic influence the use of psychotropic medications by university students and LGBTQIAPN+? A Brazilian multicenter study

**DOI:** 10.1590/0102-311XEN231323

**Published:** 2025-03-24

**Authors:** Waléria de Paula, Adriana Lúcia Meireles, Bruna Carolina Rafael Barbosa, Luiz Antônio Alves de Menezes, Clareci Silva Cardoso, Lívia Garcia Ferreira, Eulilian Dias de Freitas, Fernanda de Carvalho Vidigal, Luciana Saraiva da Silva, Luciana Neri Nobre, Elaine Leandro Machado, Helian Nunes de Oliveira, Renata Cristina Rezende Macedo do Nascimento, Glenda Nicioli da Silva

**Affiliations:** 1 Universidade Federal de Ouro Preto, Ouro Preto, Brasil.; 2 Universidade Federal de São João del-Rei, Divinópolis, Brasil.; 3 Universidade Federal de Lavras, Lavras, Brasil.; 4 Universidade Federal de Juiz de Fora, Governador Valadares, Brasil.; 5 Universidade Federal de Alfenas, Alfenas, Brasil.; 6 Universidade Federal de Uberlândia, Uberlândia, Brasil.; 7 Universidade Federal dos Vales do Jequitinhonha e Mucuri, Diamantina, Brasil.; 8 Universidade Federal de Minas Gerais, Belo Horizonte, Brasil.

**Keywords:** COVID-19, Minority Groups, Psychotropic Drugs, Mental Health, Universities, COVID-19, Grupos Minoritários, Psicotrópicos, Saúde Mental, Universidades, COVID-19, Grupos Minoritarios, Psicotrópicos, Salud Mental, Universidades

## Abstract

The COVID-19 pandemic may have had an impact on the mental health of university students and, consequently, led to the initiation on psychotropic medications or an increase on its use. This study aimed to assess the prevalence of the use of psychotropic medications among university students and evaluate the association of the use of such medicine with students who belong to minority groups. This is a cross-sectional study, in which all students enrolled in undergraduate courses at eight Brazilian public universities were invited to answer an online self-administered questionnaire. The data collection was conducted from October 2021 to February 2022. The outcome was the use of psychotropic medications and the explanatory variables were students who belong to minority groups, such as female, black, mixed-race, and other skin colors, and LGBTQIAPN+ individuals. The variables were descriptively analyzed and Pearson’s chi-squared test and multinomial logistic regression were performed. A total of 8,650 students participated in the study, of which 25.7% reported using psychotropic medications in the 30 days prior to completing the questionnaire. It was observed that among students, female (OR = 1.8; 95%CI: 1.41-2.20) and non-heterosexual (OR = 1.5; 95%CI: 1.23-1.80) had higher chances of using psychotropic medications during the COVID-19 pandemic. It was found that university students began using psychotropic medications and the association of its use with minority groups was observed.

## Resumo

A pandemia de COVID-19 pode ter impactado a saúde mental de estudantes universitários e, consequentemente, levado ao início ou ao aumento do uso de medicamentos psicotrópicos. Este estudo avaliou a prevalência do uso de medicamentos psicotrópicos entre estudantes universitários e avaliar a associação do uso dessa classe de medicamentos com estudantes de grupos minoritários. Trata-se de um estudo transversal, no qual todos os estudantes matriculados em cursos de graduação em oito universidades públicas brasileiras foram convidados. Dados foram coletados de outubro de 2021 a fevereiro de 2022, por meio de um questionário *online* autoaplicável. A variável de interesse foi o uso de medicamentos psicotrópicos e as variáveis explicativas foram estudantes pertencentes a grupos minoritários, como mulheres; negros, pardos e de outras cores de pele; e orientação sexual LGBTQIAPN+. As variáveis foram analisadas de forma descritiva, sendo realizados o teste qui-quadrado de Pearson e a regressão logística multinomial. Um total de 8.650 estudantes participaram do estudo, dos quais 25,7% relataram o uso de medicamentos psicotrópicos nos 30 dias anteriores ao preenchimento do questionário. Observou-se que, entre os estudantes, ser do sexo feminino (OR = 1,8; IC95%: 1,41-2,20) e não ser heterossexual (OR = 1,5; IC95%: 1,23-1,80) aumentaram as chances de usar medicamentos psicotrópicos durante a pandemia de COVID-19. Constatou-se que estudantes universitários iniciaram o uso de medicamentos psicotrópicos e encontrou-se associação entre esse uso e estudantes de grupos minoritários.

## Resumen

La pandemia del COVID-19 puede haber afectado a la salud mental de los estudiantes universitarios y, en consecuencia, haber llevado al inicio o aumento del uso de los psicofármacos. Este estudio evaluó la prevalencia del uso de los psicofármacos entre estudiantes universitarios y examinó la asociación de este uso con estudiantes de grupos minoritarios. Se trata de un estudio transversal, en el que se invitó a todos los estudiantes matriculados en cursos de grado en ocho universidades públicas brasileñas. Los datos se recopilaron desde octubre de 2021 hasta febrero de 2022 mediante un cuestionario en línea autoadministrado. La variable de interés fue el uso de psicofármacos; y las variables explicativas fueron estudiantes pertenecientes a grupos minoritarios, como mujeres; negros, pardos u otros colores de piel; y orientación sexual LGBTQIAPN+. Las variables se analizaron de manera descriptiva y se realizaron la prueba de chi-cuadrado de Pearson y la regresión logística multinomial. Un total de 8.650 estudiantes participaron en el estudio, de los cuales el 25,7% informó haber usado los psicofármacos a los 30 días previos a completar el cuestionario. Se observó que, entre los estudiantes, ser mujer (OR = 1,8; IC95%: 1,41-2,20) y no ser heterosexual (OR = 1,5; IC95%: 1,23-1,80) aumentaron las posibilidades de uso de psicofármacos durante la pandemia del COVID-19. Se encontró que los estudiantes universitarios empezaron a usar los psicofármacos y se encontró una asociación entre este uso y los estudiantes de grupos minoritarios.

## Introduction

The World Health Organization (WHO) emphasizes that mental health is an integral component of general health, encompassing a state of physical, emotional, psychological, and social balance ^1^. Social, economic, and environmental determinants can influence mental health ^2^ ^,^ ^3^. During crisis, such as those experienced in outbreaks, endemics, and pandemics, there is an increased fear of contracting a disease, which becomes a major stressor ^4^ ^,^ ^5^ ^,^ ^6^.

In 2019, before the COVID-19 pandemic, the worldwide prevalence of depression in women and men aged 20 to 24 years was 4.7% and 3.2%, respectively, while anxiety had a prevalence of 5.7% in women and 3.6% in men ^7^. Despite the lack of data on the prevalence of these mental disorders among university students, a systematic review involving 56,816 participants from several countries revealed a concerning situation, with prevalence rates of 26.1% for depression symptoms and 24.5% for anxiety symptoms ^8^. This scenario may have been modified by the pandemic, which led to a significant increase in stress, anxiety, and concerns regarding health, employment, finances, and uncertainties in general ^9^ ^,^ ^10^. Consequently, sales of prescription medications rose in 2020, reflecting the burden of mental health impairments related to the pandemic ^11^ ^,^ ^12^ ^,^ ^13^. Psychotropic medication use can differ between university students and the general population, influenced by factors like age, academic environment, workload, stress levels, and mental health prevalence. Studies show the rates of use of psychotropic medications among university students increased from 12.7% to 30.4% and may even surpass those observed in the general population ^14^ ^,^ ^15^ ^,^ ^16^.

University students face academic pressure, expectations, transitions, and emotional demands during their studies ^17^. For minority groups, including women, black and mixed-race people, and LGBTQIAPN+ individuals, the COVID-19 pandemic may have intensified these challenges ^18^ ^,^ ^19^ ^,^ ^20^ ^,^ ^21^ ^,^ ^22^ ^,^ ^23^. These groups often face barriers to mental health services, such as a shortage of professionals and discrimination ^24^ ^,^ ^25^. The pandemic worsened these issues by straining health systems and limiting in-person services, which may have increased reliance on medication ^26^ ^,^ ^27^. Because psychological support and on-site therapy were often interrupted, some countries temporarily eased restrictions on psychotropic medications ^28^ ^,^ ^29^. Inadequate social and family support may further hinder treatment effectiveness and increase negative mental health outcomes for these minorities ^30^ ^,^ ^31^. 

Studies on pharmacoepidemiology are of paramount importance in collecting data on the effectiveness and safety of medications use and prescription profile, as well as factors associated with use, among others ^32^. Although studies on psychotropic medication use during the pandemic exist ^33^ ^,^ ^34^ ^,^ ^35^, data on university students, especially minority groups, are lacking. This study hypothesizes the COVID-19 pandemic may have increased the use of psychotropic medication among university students, potentially via self-medication, despite the Brazilian regulation under *Federal Ordinance n. 344* ^36^, 1998. Additionally, minority students - such as women, black, mixed-race, and LGBTQIAPN+ individuals - may have used these medications more than other students during the pandemic.

Therefore, this study aimed to assess the prevalence of the use of psychotropic medications and self-medication, as well as associations with minority groups, comparing pre-pandemic and pandemic periods among students at Federal Higher Education Institutions (IFES) in Brazil.

## Methods

### Study design and population

This cross-sectional study is part of the survey *Symptoms of Anxiety and Depression Disorders among University Students in Minas Gerais: Multicenter Study* (*Project on Anxiety and Depression in University Students* - PADu-multicenter), whose population are undergraduate students of eight public universities: Federal University of Ouro Preto (UFOP, acronym in Portuguese), Federal University of Minas Gerais (UFMG, acronym in Portuguese), Federal University of Juiz de Fora (UFJF, acronym in Portuguese), Federal University of São João del-Rei (UFSJ, acronym in Portuguese), Federal University of Lavras (UFLA, acronym in Portuguese), Federal University of Alfenas (UNIFAL, acronym in Portuguese), Federal University of Jequitinhonha and Mucuri Valleys (UFVJM, acronym in Portuguese), and Federal University of Uberlândia (UFU, acronym in Portuguese).

All students aged over 18 years, enrolled in undergraduate courses during the second semester of 2021, were invited to participate in the PADu-multicenter, totaling 118,828 students. A list of students’ email addresses was provided by the university itself so that researchers could send out invitations to participate in the survey. In other cases, the invitation was automatically sent via the university’s own communication system. The PADu-multicenter dataset included valid responses from 8,650 students. All students were invited to complete the survey, so no initial sample size calculation was performed. Posthoc power analysis showed satisfactory results (normal approximation > 98%). With a 7.3% response rate, the sample size was sufficient to detect significant associations, ensuring robust and reliable findings. For more details, see Barbosa et al. ^37^.

### Data collection

The research was publicized on the official websites and social networks of the participating institutions, as well as in research groups, centers, and academic departments. Data collection was conducted from October 2021 to February 2022 via an online and self-administered questionnaire, available on Google Forms (https://docs.google.com/forms/u/0/), with questions regarding sociodemographic and academic characteristics, lifestyle habits, and health conditions. 

All procedures were performed according to the Brazilian guidelines and standards for research involving human beings of the *Declaration of Helsinki* and approved by the Research Ethics Committee of the participating universities (CAAE from the coordinating center - UFOP: 43027421.3.1001.5150).

### Study variables

The outcome variable was the use of psychotropic medications (anxiolytics and sedatives, antidepressants, antipsychotics, psychomotor stimulants, psychomimetics, and those with cognitive enhancing activity). Such use was determined by self-report, based on the answers to the questions: (1) In the last 30 days, did you use any medication?; (2) Mention the name of the medication you are taking or have used in the last 30 days; and (3) Did you start using the medication after the onset of the pandemic? For questions one and three the answer options were no/yes. The second was an open-ended question. The reported medications were grouped into classes according to the third (pharmacological class) and fifth (chemical substance) levels of the Anatomical Therapeutic Chemical (ATC classification) ^38^.

Subsequently, the participants were classified into four groups: those who do not use psychotropic medication; those who reported using some psychotropic medication before the COVID-19 pandemic; those who started using psychotropic medications during the pandemic; and participants who were already using them previous to the pandemic and started using new psychotropic medications during the pandemic.

The explanatory variables were students who belong to minority groups: female; non-white skin color, such as black, mixed-race and others (Asians, Indigenous people); and non-heterosexual, (LGBTQIAPN+).

Sociodemographic, lifestyle habits, and health conditions variables were used to characterize the sample, such as age (18-22 years old or > 22 years old), marital status (single, married, stable union, widowed, or divorced), type of housing (alone, with family or with friends or colleagues in an apartment, house, or dormitory), family income (up to 4 minimum wages or more than 4 minimum wages in Brazil), knowledge area (Life Sciences, Exact Sciences, Human and Social Sciences, or Applied Sciences), routine of academic activities (fully remote, hybrid, or on-site), alcohol consumption (no/yes), smoking (no/yes), practice of physical activity (no/yes), overweight (no/yes), COVID-19 infection (no, I think so (not confirmed by test), yes), diagnosis of chronic illness (no/yes), suicidal ideation (no/yes), anxiety (normal/mild, moderate, or severe/extremely severe), depression (normal/mild, moderate, severe, or extremely severe), and stress (normal/mild, moderate, or severe/extremely severe).

The variables were obtained from questions adapted from national surveys, such as the *Brazilian Household Budget Survey* ^39^ and *Brazilian National Survey of Health* ^40^. The variable overweight (no/yes) was self-reported by the students, based on the body mass index, and subsequently categorized regarding age group, according to the reference values recommended by WHO for adolescents and adults ^41^. The assessment of anxiety, depression, and stress symptoms was performed using the *Depression Anxiety Stress Scale-21* (DASS-21), adapted and validated for the Brazilian Portuguese by Vignola & Tucci ^42^. The DASS-21 enables a summation, which is later multiplied by two, generating scores that allow for classifying symptoms into five levels: “normal”, “mild”, “moderate”, “severe” and “extremely severe”. In this study, symptoms were reclassified into three categories: “normal/mild”, “moderate” and “severe/extremely severe”.

### Statistical analysis

The consistency of the collected data and the database coding was performed using Microsoft Excel 2013 (https://products.office.com/). Statistical analyses were performed using Stata software, version 13.0 (https://www.stata.com). Firstly, descriptive analysis was conducted using frequency distribution and bivariate analysis using Pearson’s chi-squares test. The covariates related to the use of psychotropic medications at p < 0.050 in the bivariate analysis were included as adjustments in the multivariate analysis.

For the multivariate analysis, three explanatory variables were considered: sex, skin color, and sexual orientation. A multinomial logistic regression was performed, which enabled odds ratios (OR) calculation considering an outcome with more than two categories and each category was compared to the reference category in a single processing. The reference category was non-use of psychotropic medications, which was compared to three categories: use of psychotropic medications in the period before the pandemic; use of psychotropic medications during the pandemic; previous use of psychotropic medications and the use of new ones during the pandemic. Notably, 95% confidence intervals (95%CI) were used.

## Results

The sample population was composed of 8,650 students. It was observed that most were female (65.7%), self-declared white (55.4%), heterosexual (66.1%), aged from 18 to 22 years (54.2%), and had a mean age of 23.9 years (standard deviation - SD ±6.3). Severe or extremely severe symptoms of anxiety and depression affected 43.5% and 44.8% of students, respectively (Table 1).

**Table 1:** Descriptive and bivariate analyses of sociodemographic characteristics, lifestyle habits, and health conditions of university students according to the use of psychotropic medications during the COVID-19 pandemic. PADu-multicenter, 2022 (N = 8,650).

Variables	Total	No use of psychotropic medications	Use of psychotropic medications separated by period of use			p-value
			Before the pandemic	After the onset of the pandemic	Previous and addition after the onset of the pandemic	
	n (%)	n (%)	n (%)	n (%)	n (%)	
Sociodemographic						
Sex [n = 8,615]						< 0.001
Male	2,955 (34.3)	2,371 (37.0)	166 (29.8)	282 (28.5)	136 (20.7)	
Female	5,660 (65.7)	4,038 (63.0)	392 (70.2)	708 (71.5)	522 (79.3)	
Skin color [n = 8,474]						0.001
White	4,694 (55.4)	3,409 (54.1)	329 (59.7)	569 (57.9)	387 (60.2)	
Mixed-race, black and others	3,780 (44.6)	2,888 (45.9)	222 (40.3)	414 (42.1)	256 (39.8)	
Sexual orientation						< 0.001
Heterosexual	5,714 (66.1)	4,457 (69.3)	364 (64.8)	552 (55.4)	341 (51.5)	
LGBTQIAPN+	2,936 (33.9)	1,973 (30.7)	198 (35.2)	444 (44.6)	321 (48.5)	
Age (years)						< 0.001
18-22	4,687 (54.2)	3,665 (57.0)	199 (35.4)	528 (53.0)	295 (44.6)	
> 22	3,963 (45.8)	2,765 (43.0)	363 (64.6)	468 (47.0)	367 (55.4)	
Marital status [n = 8,582]						0.145
Single	7,775 (90.6)	5,787 (90.7)	491 (88.5)	912 (91.8)	585 (89.7)	
Married, stable union, widowed, divorced	807 (9.4)	595 (9.3)	64 (11.5)	81 (8.2)	67 (10.3)	
Type of housing						< 0.001
Alone	731 (8.2)	498 (7.7)	60 (10.7)	84 (8.4)	71 (10.7)	
With famiy	6,602 (76.3)	4,994 (77.7)	403 (71.7)	727 (73.0)	478 (72.2)	
With friends or colleagues in an apartment, house or dormitory	1,335 (15.5)	938 (14.6)	99 (17.6)	185 (18.6)	113 (17.1)	
Family income (minimum wages) * [n = 8,090]						< 0.001
Up to 4	4,030 (49.8)	3,009 (50.1)	270 (50.7)	497 (54.9)	254 (41.2)	
More than 4	4,060 (50.2)	2,991 (49.9)	263 (49.3)	443 (47.1)	363 (58.8)	
Field of knowledge						< 0.001
Life Sciences	3,416 (39.5)	2,704 (42.0)	180 (32.0)	344 (34.5)	188 (28.4)	
Exact Sciences	2,731 (31.6)	1,971 (30.7)	206 (36.7)	319 (32.0)	235 (35.5)	
Human and Social Sciences, or Applied Sciences	2,503 (28.9)	1,755 (27.3)	176 (31.3)	333 (33.5)	239 (36.1)	
Routine of academic activities						0.010
Fully remote	7,711 (89.2)	5,779 (89.9)	496 (88.3)	874 (87.7)	568 (85.8)	
Hybrid	838 (9.7)	582 (9.0)	60 (10.7)	114 (11.5)	82 (12.4)	
On-site	95 (1.1)	69 (1.1)	6 (1.0)	8 (0.8)	12 (1.8)	
Lifestyle habits						
Alcohol consumption						0.073
No	3,248 (37.5)	2,411 (37.5)	238 (42.4)	359 (36.0)	240 (36.3)	
Yes	5,402 (62.5)	4,019 (62.5)	324 (57.6)	637 (64.0)	422 (63.7)	
Smoking						< 0.001
No	7,295 (84.3)	5,537 (86.1)	451 (80.3)	783 (78.6)	524 (79.2)	
Yes	1,355 (15.7)	893 (13.9)	111 (19.7)	213 (21.4)	138 (20.8)	
Practice of physical activity						0.001
No	3,113 (36.0)	2,268 (35.3)	245 (43.6)	351 (35.2)	249 (37.6)	
Yes	5,537 (64.0)	4,162 (64.7)	317 (56.4)	645 (64.8)	413 (62.4)	
Health conditions						
Overweight						< 0.001
No	5,604 (65.1)	4,304 (67.2)	312 (55.9)	631 (63.6)	357 (54.4)	
Yes	3,005 (34.9)	2,099 (32.8)	246 (44.1)	361 (36.4)	299 (45.6)	
COVID-19 infection [n = 8,615]						0.014
No	6,390 (74.2)	4,765 (74.4)	425 (76.0)	705 (70.9)	495 (75.1)	
I think so (not confirmed by test)	921 (10.7)	708 (11.1)	46 (8.2)	109 (11.0)	58 (8.8)	< 0.001
Yes	1,304 (15.1)	930 (14.5)	88 (15.8)	180 (18.1)	106 (16.1)	
Diagnosis of chronic condition						< 0.001
No	2,288 (26.5)	2,234 (34.7)	10 (1.8)	35 (3.5)	9 (1.4)	
Yes	6,362 (73.5)	4,196 (65.3)	552 (98.2)	961 (96.5)	653 (98.6)	
Suicidal ideation [n = 4,443]						< 0.001
No	4,605 (55.6)	3,862 (62.6)	203 (37.7)	340 (35.7)	200 (31.8)	
Yes	3,679 (44.4)	2,304 (37.4)	335 (62.3)	611 (64.3)	429 (68.2)	
Anxiety						< 0.001
Normal/Mild	3,486 (40.3)	2,947 (45.8)	149 (26.5)	234 (23.5)	156 (23.6)	
Moderate	1,405 (16.2)	1,068 (16.6)	95 (16.9)	140 (14.1)	102 (15.4)	
Severe/Extremely severe	3,759 (43.5)	2,415 (37.6)	318 (56.6)	622 (62.4)	404 (61.0)	
Depression						< 0.001
Normal/Mild	3,201 (37.0)	2,697 (42.0)	133 (23.7)	215 (21.6)	156 (23.6)	
Moderate	1,574 (18.2)	1,152 (17.9)	112 (19.9)	180 (18.1)	130 (19.6)	
Severe/Extremely severe	3,875 (44.8)	2,581 (40.1)	317 (56.4)	601 (60.3)	376 (56.8)	
Stress						< 0.001
Normal/Mild	3,679 (42.5)	3,046 (47.4)	181 (32.2)	260 (26.1)	192 (29.0)	
Moderate	1,467 (17.0)	1,100 (17.1)	93 (16.6)	172 (17.3)	102 (15.4)	
Severe/Extremely severe	3,504 (40.5)	2,284 (35.5)	288 (51.2)	564 (56.6)	368 (55.6)	

Note: the test used to evaluate associations between the four groups was the chi-square test.

* Value of the minimum wage in force in Brazil during the collection period: BRL 1,100.

The use of at least one medication in the 30 days prior to completing the questionnaire, regardless of the pharmacological class, was reported by 4,629 participants (53.5%), with a mean of 1.9 medications (SD ±1.3). The prevalence of psychotropic medications use was 25.7% (95%CI: 24.8-26.6) regarding the total sample.

**Figure 1:**
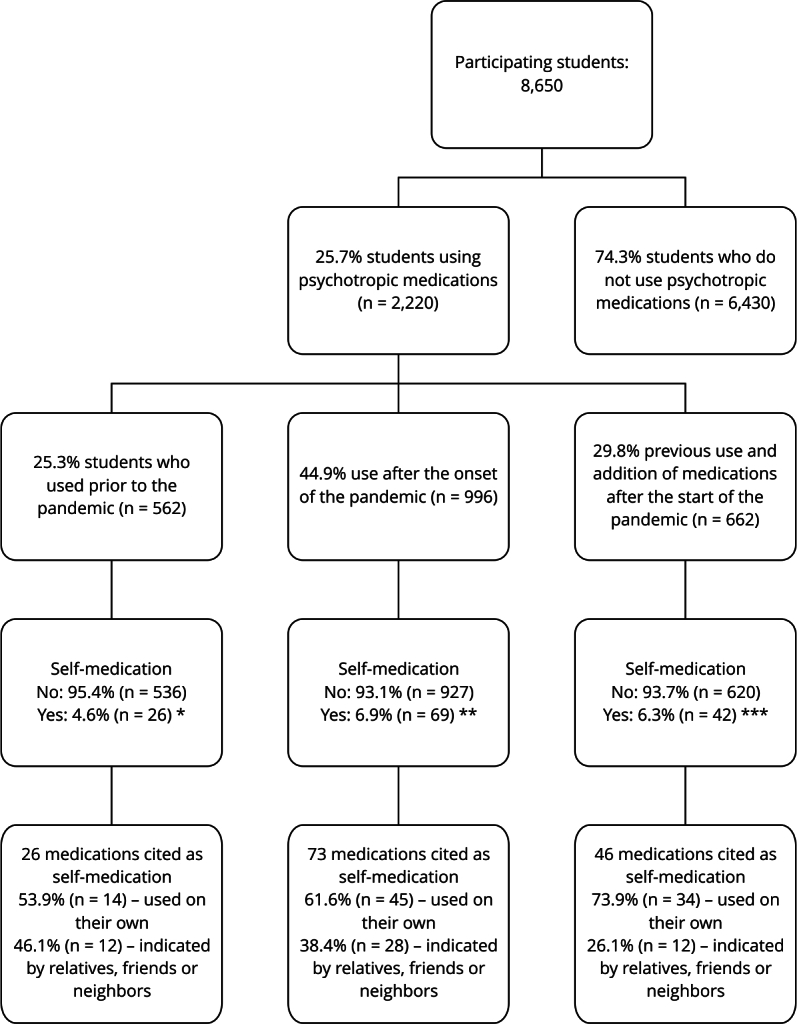
Use of psychotropic medications by the university population participating in the PADu-multicenter, 2022 (n = 2,220), during the COVID-19 pandemic.

Note: 145 medications were cited for self-medication, and more than one medication could have been cited per student. In parentheses is the number of citations for each medication. * Bromazepam (1), buspirone (1), mebeverine (1), venlafaxine (1), cannabidiol (2), escitalopram (2), fluoxetine (2), melatonin (2), alprazolam (3), clonazepam (3) , methylphenidate (3), valerian (5); ** 5-Hydroxytryptophan (1), alprazolam (1), amitriptyline (1), bromazepam (1), buspirone (1), flurazepam (1), lisdexamfetamine (1), melatonin (1), tryptophan (1), venlafaxine (1), bupropion (2), escitalopram (2), sertraline (2), diazepam (4), fluoxetine (4), methylphenidate (4), zolpidem (8), valerian (10), clonazepam (27); *** Amitriptyline (1), betahistine (1), bromazepam (1), caffeine (1), escitalopram (1), flunarizine (1), fluoxetine (1), lisdexamfetamine (1), melatonin (1), nortriptyline (1), risperidone (1), gamma-aminobutyric acid (2), bupropion (2), methylphenidate (2), valerian (6), clonazepam (7), alprazolam (8), zolpidem (8).

Among students using psychotropic medications, most started using them during the COVID-19 pandemic (44.9%), followed by those who were already using them and added new medications from the same pharmacological class during the pandemic (29.8%) and, finally, those who were already using them before the pandemic and maintained this use (25.3%). Moreover, 137 (6.2%) cases of self-medication were identified (Figure 1).

Regarding the number of medications used, 3,741 psychotropic medications were reported by the students, who could use more than one medication or class at the same time. Antidepressants (N06A) were the most used, being cited by 38.5% (n = 1,782) of the students who reported using some medication (n = 4,629). In addition, 11.3% (n = 524) reported the use of antiepileptics (N03A); 6.4% (n = 297) of antipsychotics (N05A); 5.4% (n = 251) of hypnotics and sedatives (N05C); 4.6% (n = 215) of anxiolytics (N05B), and 3.9% (n = 179) psychostimulants, agents used for attention deficit hyperactivity disorder (ADHD) and nootropics (N06B). Table 2 shows the number of medications citations by category and ranking of the most cited medications by the students, respectively. 

**Table 2:** Number of medications reported by university students, according to the pharmacological category (ATC level 3) and ranking of the five most cited psychotropic medications (ATC level 5) by university students. PADu-multicenter, 2022.

ATC code/Class	Medication	n
N06A/Antidepressants		2,105
N06AB10	Escitalopram	410
N06AB06	Sertraline	372
N06AX16	Venlafaxine	212
N06AX23	Desvenlafaxine	199
N06AB03	Fluoxetine	186
N03A/Antiepiletics		580
N03AE01	Clonazepam	265
N03AX11	Topiramate	98
N03AX09	Lamotrigine	68
N03AG01	Valproic acid	59
N03AX16	Pregabalin	39
N05A/Antipsychotics		347
N05AH04	Quetiapine	134
N05AN01	Lithium carbonate	106
N05AX08	Risperidone	34
N05AX12	Aripiprazole	19
N05AH03	Olanzapine	13
N05C/Hypnotics and sedatives		258
N05CF02	Zolpidem	167
N05CM09	Valerian	47
N05CH01	Melatonin	23
N05CF04	Eszopiclone	9
N05CM05	Scopolamine	5
N05B/Anxiolytics		232
N05BA12	Alprazolam	132
N05BE01	Buspirone	44
N05BA01	Diazepam	22
N05BA08	Bromazepam	16
N05BB01	Hydroxyzine	9
N06B/Psychostimulants, ADHD agents and nootropics		182
N06BA04	Methylphenidate	125
N06BA12	Lisdexamfetamine	48
N06BA07	Modafinil	6
N06BC01	Caffeine	2
N06BX03	Piracetam	1
N07C/Others (anticholinergic agents, dopaminergic agents, psycholeptics and psychoanaleptics in combination, parasympathomimetics, antivertigo, other drugs for the nervous system)		37
N07CA03	Flunarizine	7
N07BB04	Naltrexone	7
N07CA01	Betahistine	6
N04BC06	Cabergoline	5
N04AA02	Biperiden	3

ADHD: attention deficit hyperactivity disorder; ATC: Anatomical Therapeutic Chemical.

Table 3 shows the results of the multinomial logistic regression between minority groups and the use of psychotropics. Compared to male students, female students had a greater chance of starting and increasing the use of psychotropic medications during the pandemic (OR = 1.8; 95%CI: 1.41-2.20). Those who declared themselves mixed-race, black and others had lower chances of starting (OR = 0.8; 95%CI: 0.68-0.92) or increasing (OR = 0.7; 95%CI: 0.56-0.81) the use of psychotropic medications when compared to those who declared themselves white. And, finally, LGBTQIAPN+ students were more likely to have started (OR = 1.3; 95%CI: 1.09-1.48) or increased (OR = 1.5; 95%CI: 1.23-1.80) the use of psychotropic medications during the pandemic compared to heterosexual students.

**Table 3:** Multinominal regression between minority groups and the use of psychotropic medications, during the COVID-19 pandemic, unadjusted and adjusted analysis. PADu-multicenter, 2022 (N = 8,650).

Variables	Psychotropic medications use		
	Use prior to the pandemic	Use after the onset of the pandemic	Previous use and addition after the onset of the pandemic
	OR (95%CI)	OR (95%CI)	OR (95%CI)
Unadjusted analysis			
Sex [n = 8,615]			
Male	1.00	1.00	1.00
Female	1.40 (1.15-1.67) *	1.50 (1.27-1.71) **	2.20 (1.85-2.74) **
Skin color [n = 8,474]			
White	1.00	1.00	1.00
Mixed-race, black and others	0.80 (0.67-0.95) ***	0.90 (0.74-0.98) ***	0.80 (0.66-0.92) *
Sexual orientation			
Heterosexual	1.00	1.00	1.00
LGBTQIAPN+	1.2 (1.02-1.47) ***	1.80 (1.59-2.08) **	2.10 (1.81-2.50) **
Adjusted analysis ^#^			
Sex [n = 8,615]			
Male	1.00	1.00	1.00
Female	1.10 (0.86-1.34)	1.10 (0.94-1.33)	1.80 (1.42-2.24) **
Skin color [n = 8,474]			
White	1.00	1.00	1.00
Mixed-race, black and others	0.70 (0.57-0.85) **	0.80 (0.68-0.92) *	0.70 (0.62-0.91) *
Sexual orientation			
Heterosexual	1.00	1.00	1.00
LGBTQIAPN+	0.90 (0.70-1.07)	1.30 (1.07-1.48) *	1.40 (1.35-1.98) **

95%CI: 95% confidence interval; OR: odds ratio.

* p < 0.01;

** p < 0.001;

*** p < 0.05;

^#^ Adjusted values for all covariates that had a p < 0.050 in the bivariate analysis (age, type of housing, family income, knowledge area, routine of academic activities, smoking, practice of physical activity, overweight, COVID-19 infection, diagnosis of chronic illness, suicidal ideation, anxiety, depression, stress). Non-use of psychotropic medications was used as a comparison parameter.

## Discussion

This study found a high prevalence of the use of psychotropic medication among Brazilian university students, with over a quarter reporting usage. Many students started or increased the use of these medications during the pandemic, reflecting the impact of this period. Instances of self-medication with prescription-only medications were noted. Female and LGBTQIAPN+ students were more likely to start or increase the use of psychotropic medication during the pandemic.

Prevalence studies are crucial for understanding students’ mental health, guiding policies, facilitating interventions, reducing stigma, and directing future research ^43^ ^,^ ^44^. This research is essential for providing the support students need for academic and personal success.

A 2019 study on Brazilian medical students reported lower use of psychotropic before the pandemic ^14^. Another study indicated an increased use of psychotropic among younger people (18-25 years), which includes most university students ^13^. Therefore, a rise in psychotropic use during the pandemic was anticipated, driven by fear and uncertainty. Studies from other Brazilian states reported the use of psychotropic medications rates ranging from 10.1% to 37.3% ^45^ ^,^ ^46^ ^,^ ^47^.

Pre-pandemic studies showed lower rates of mental disorders than our study for both the general population and university students ^48^ ^,^ ^49^ ^,^ ^50^ ^,^ ^51^. Students are particularly vulnerable due to academic pressures and various demands ^52^. Sabião et al. ^53^ found lower rates of symptoms of mental disorders in adults and older adults during the pandemic than this study, highlighting the significant emotional and mental challenges students faced, including social isolation, routine changes, and health concerns ^54^. The high use of psychotropic medications among students reflects this increased mental load.

The increased use of psychotropic medications during the pandemic is not inherently negative. These medications are prescribed for conditions such as depression, anxiety, bipolar disorder, and sleep disorders ^55^, and can be necessary and beneficial for managing mental health. The most cited types include antidepressants, antiepileptics, antipsychotics, and hypnotics, of which escitalopram, sertraline, clonazepam, and zolpidem being frequently used, consistent with other studies on Brazilian students ^47^ ^,^ ^56^. A study in Spain also noted a significant rise in antidepressant use among younger people after the onset of the pandemic ^57^.

Some psychotropic medications, like clonazepam, initially developed as anticonvulsants, are now widely used as anxiolytics, particularly in Brazil ^58^. Anticonvulsants like valproic acid, carbamazepine, and lithium are also commonly used as mood stabilizers for bipolar disorders ^59^. Understanding these distinctions is crucial for interpreting psychotropic use data, highlighting their psychiatric rather than neurological application among university students.

Cases of self-medication were identified in this study, and this medication category is regulated by *Federal Ordinance n.* 344 ^36^ of 1998 that approves the technical regulation on substances and medications subject to special control. Among the total participants, 14.3% reported self-medication. When considering only students who reported using psychotropic medications, the percentage is 1.6%, and clonazepam, alprazolam and zolpidem were the most cited. A study ^60^ with Colombian medical students found a 39.5% rate of psychotropic medication use, including self-medication with herbal products. The most cited medications were fluoxetine, zolpidem, and trazodone ^60^.

The discrepancy between the high prevalence of severe anxiety and depression symptoms and the lower use of psychotropic medications among university students may be due to factors like limited access to mental health care and stigma. During the COVID-19 pandemic, barriers such as virtual care and limited availability of healthcare providers worsened this issue ^54^ ^,^ ^61^. Students may underdiagnose or underestimate their symptoms, affected by pandemic-related stressors like isolation and academic uncertainty ^19^ ^,^ ^62^. Social and cultural stigma around mental health and the use of medication also discourage students from seeking treatment, leading them to rely on alternatives or self-manage of symptoms ^63^. These factors explain why many students with severe symptoms avoid psychotropic medications, highlighting the complexities of mental health treatment during a global crisis.

Self-medication can be dangerous and lead to undesirable consequences such as addiction, compulsion, and adverse events ^55^. The use of psychotropic medications needs to be monitored by health professionals, who can assess the need, make appropriate prescriptions, and provide continuous monitoring and support ^64^. 

In our findings, most students who belong to minority groups were more likely to use psychotropic medications during the COVID-19 pandemic compared to their peers, except those identifying as black, mixed-race and others.

The designation of a group as a minority is based not only on numbers but also on power dynamics, inequalities, and discrimination. Minority groups often have diminished access to resources and representation, leading to social and economic disadvantages ^65^ ^,^ ^66^. The pandemic added stress, anxiety, depression, and adaptation challenges may have prompted seeking professional help and considering medication as part of treatment ^67^.

Women tend to report higher levels of anxiety and depression, which is often worsened by social and academic pressures, leading to increased use of psychotropic medications for symptom management ^57^ ^,^ ^68^. Female students deal daily with different stressors, such as gender discrimination, social inequalities, and social and family pressures, in addition to specific challenges related to female health. These experiences, added to the stressors of the pandemic, may have contributed to a greater chance of developing mental health disorders and the need for psychotropic treatment during the pandemic ^57^ ^,^ ^68^ ^,^ ^69^. In addition, women may be more likely to seek health services and express their emotions compared to men ^70^.

Previous studies show LGBTQIAPN+ individuals are more likely to experience depression, anxiety, suicide attempts, and substance abuse compared to heterosexuals. Factors like discrimination, stigma, lack of support, marginalization, and violence contribute to these challenges ^21^ ^,^ ^22^ ^,^ ^71^ ^,^ ^72^. Social distancing has reduced social circles and support, possibly leading to increased use of psychotropic medications.

The increased use of psychotropic medications among university students, particularly women and LGBTQIAPN+ individuals, may stem from factors like mental disorder prevalence, access to care, family support, and discrimination. Research indicates that women and LGBTQIAPN+ individuals report higher anxiety and depression, leading to greater medication use ^73^ ^,^ ^74^. LGBTQIAPN+ students without family support face higher mental health risks and rely more on medication, probably driven because of social isolation and lower family acceptance ^75^ ^,^ ^76^. The pandemic has exacerbated these issues, increasing the use of psychotropic medication as a coping mechanism ^77^.

Although the literature lacks studies showing differences in mental health between white students and racial minorities regarding the use of psychotropic medication during the pandemic, some studies found that white students reported more symptoms of mental disorders potentially leading to medication use ^20^ ^,^ ^78^. Previous research indicates that white skin color is a remarkable sociodemographic factor that contributes to unprescribed use of sedatives/hypnotics among young adults ^79^ ^,^ ^80^. Black university students may use less psychotropic medications due to barriers to access mental health services, historical distrust of healthcare, and cultural stigma, resulting in treatment disparities ^81^ ^,^ ^82^. Our findings suggest social, economic, and health factors - such as income, education, and access to housing and health service - may contribute to disparities in medication use among white students.

This study must be considered in light of its limitations and strengths. Although this study uses a convenience sample of university students, the considerable number of participants offers advantages such as increased statistical power and better representation of demographic subgroups ^83^. Online data collection faces challenges such as limited internet access, high non-response rates, selection bias, and platform familiarity. Despite these issues, online studies have been proved to be crucial, and often the only option, for research during the COVID-19 pandemic ^84^. Self-reported information was used, with potential memory and information bias. Although the study design does not enable inferring causality, the objective of knowing the prevalence of the use of psychotropic medication initiated during the pandemic was met. Another limitation of our study is the measurement of the use of psychotropic medication, which relied on a dichotomous question (no/yes) about use in the 30 days before the survey and whether it began after the pandemic. Since data collection started in October 2021, changes in medication patterns due to immediate pandemic stressors might not be fully captured. However, the 30-day window offers insight into the ongoing impact of the pandemic on the use of psychotropic medication. The influence of the use of psychotropic medication could not be clearly attributed solely to the COVID-19 pandemic or the transition to university life. This is especially relevant for students who began university during the pandemic. Future studies should separate these factors for a more accurate analysis.

As strengths, the information was obtained in a critical period in which remote teaching at universities was taking place due to the COVID-19 pandemic, which allowed progress in elucidating how the pandemic may have affected the mental health of the university population in minority groups and a survey of the use of psychotropic medications by students.

Studies on the use of medications are very scarce in the literature and our findings reinforce how important and necessary it is to worry about the university population, mostly youth, and who will be professionals in the future.

Most students used psychotropic medications and those who were female and LGBTQIAPN+ had an increased chance of starting or increasing the use of psychotropic medications during the pandemic, which reflects a mentally ill population that sought relief for symptoms - mainly anxiety, depression, and insomnia - in pharmacotherapy. 

As the promotion of mental health is essential for the general improvement of people’s health and quality of life, students must have access to psychological support. Universities and educational institutions should offer student counseling services and other forms of support to help students who are experiencing emotional and mental difficulties. Such actions can help to reduce the cases of evasion and suspension.
